# CRKL regulates alternative splicing of cancer-related genes in cervical cancer samples and HeLa cell

**DOI:** 10.1186/s12885-019-5671-8

**Published:** 2019-05-27

**Authors:** Qingling Song, Fengtao Yi, Yuhong Zhang, Daniel K. Jun Li, Yaxun Wei, Han Yu, Yi Zhang

**Affiliations:** 1 Department of Oncology and Radiotherapy, Wuhan General Hospital of Guangzhou Military Command, Wuhan, 430070 Hubei Province China; 2 Laboratory of Human Health and Genome Regulation, Wuhan, 430075 Hubei China; 3 Center for Genome Analysis, ABLife Inc, Wuhan, 430075 Hubei China; 4 Department of Biology and Biotechnology, School of Chemistry, Chemical Engineering and Life Science, Wuhan University of Technology, Wuhan, 430070 Hubei China

**Keywords:** CRKL, RNA-seq, Alternative splicing, Cervical carcinoma, Tumorigenesis

## Abstract

**Background:**

Aberrant spliced isoforms are specifically associated with cancer progression and metastasis. The cytoplasmic adaptor *CRKL* (v-crk avian sarcoma virus CT10 oncogene homolog-like) is a CRK like proto-oncogene, which encodes a SH2 and SH3 (src homology) domain-containing adaptor protein. CRKL is tightly linked to leukemia via its binding partners BCR-ABL and TEL-ABL, upregulated in multiple types of human cancers, and induce cancer cell proliferation and invasion. However, it remains unclear whether signaling adaptors such as CRKL could regulate alternative splicing.

**Methods:**

We analyzed the expression level of *CRKL* in 305 cervical cancer tissue samples available in TCGA database, and then selected two groups of cancer samples with CRKL differentially expressed to analyzed potential CRKL-regulated alternative splicing events (ASEs). CRKL was knocked down by shRNA to further study CRKL-regulated alternative splicing and the activity of SR protein kinases in HeLa cells using RNA-Seq and Western blot techniques. We validated 43 CRKL-regulated ASEs detected by RNA-seq in HeLa cells, using RT-qPCR analysis of HeLa cell samples and using RNA-seq data of the two group of clinical cervical samples.

**Results:**

The expression of *CRKL* was mostly up-regulated in stage I cervical cancer samples. Knock-down of *CRKL* led to a reduced cell proliferation. CRKL-regulated alternative splicing of a large number of genes were enriched in cancer-related functional pathways, among which DNA repair and G2/M mitotic cell cycle, GnRH signaling were shared among the top 10 enriched GO terms and KEGG pathways by results from clinical samples and HeLa cell model. We showed that CRKL-regulated ASEs revealed by computational analysis using ABLas software in HeLa cell were highly validated by RT-qPCR, and also validated by cervical cancer clinical samples.

**Conclusions:**

This is the first report of CRKL-regulation of the alternative splicing of a number of genes critical in tumorigenesis and cancer progression, which is consistent with CRKL reported role as a signaling adaptor and a kinase. Our results underline that the signaling adaptor CRKL might integrate the external and intrinsic cellular signals and coordinate the dynamic activation of cellular signaling pathways including alternative splicing regulation.

**Electronic supplementary material:**

The online version of this article (10.1186/s12885-019-5671-8) contains supplementary material, which is available to authorized users.

## Background

Cervical cancer, frequently leading to death, is one of the most common gynecological malignancies among women globally [1]. Fortunately, the incidence of advanced cervical cancer and cervical-cancer mortality has been dramatically reduced through screening for human papillomavirus (HPV) instead of a single conventional cytological test or visual inspection [2]. Although efficient diagnosis during precancerous and early stages of cervical cancer is pivotal for the effective cure of cervical cancer [3–5], the effectiveness of cervical cancer treatments has not been improved significantly over the past decades [6–8]. The overall cervical-cancer incidence and mortality increased steadily from 1991 to 2013, which has been predicted to continue in the future [9]. Therefore, it is very important to identify molecular markers and therapeutic targets to improve the effectiveness of cervical cancer treatment.

Alternative splicing is one of the key molecular mechanism contributing to the biological functional complexity of the human genome [10]. The alternative processing of primary RNA transcripts of individual mammalian genes produces various mRNA and protein isoforms which have related, distinct or even opposing functions [11–13]; These include both widespread homeostatic activities and cell-type-specific functions [14]. It was reported that transcripts from ~ 95% of multiexon genes are alternatively spliced. In major human tissues, there are about 100,000 intermediate- to high-abundance alternative splicing events [15]. In the past few years, emerging data suggest that the cancer progression and metastasis are specifically associated with a plethora of mRNA isoforms [16–21]. Noncanonical and cancer-specific mRNA transcripts produced by the aberrant splicing can lead to loss of function of tumor suppressors or activator of oncogenes and cancer pathways [21]. These cancer-specific isoforms may represent attractive cancer therapeutic targets. Recently, it was reported that alternative splicing regulates cervical cancer oncogenesis via miL1RAP-NF-κB-CD47 axis, indicative of an attractive therapeutic target for treatment of cervical cancer [22].

The center of tumorigenesis is the activation of various signal transduction pathways, and key kinases in these pathways represent a large class of effective therapeutic targets [23–26]. For example, a wide range of epithelial cancers have aberrant activation of EGFR signaling by overexpression or mutation, and targeting EGFR signaling network thus represents a rational for novel treatment approaches [27–29]. Overexpression of components of cAMP/CREB pathway is related to a subset of human carcinomas, indicating a potential therapeutic strategies for this group of tumors [25]. The activation of the PI3K/Akt pathway is associated with incomplete metabolic response in cervical cancer and therefore represents a therapeutic target in cervical cancer [26].

Signaling adaptors connect the activated cell-surface receptors with down-stream effectors (kinases) in the signaling pathways, via their domains and motifs mediating molecular interactions [30–33]. These adaptors are increasingly recognized as coordinators of the dynamic activation of signaling pathways in response to both external and intrinsic stimuli and/or changes [34–38]. The cytoplasmic adaptor *CRKL* (v-crk avian sarcoma virus CT10 oncogene homolog-like) is a CRK like proto-oncogene, which belongs to Crk family adaptors and encodes a SH2 and SH3 (src homology) domain-containing adaptor protein [32]. This adaptor can form multi-protein complexes by selective interaction with a number of adaptor proteins, including paxillin (substrate for several normal and oncogenic tyrosine kinases), p130CAS (130 kDa Crk associated kinase substrate) and p120 c-cbl (maintenance of T-cell non-responsiveness), as well as insulin receptor substrate proteins (IRS), STAT5 and PI3K (phosphatidylinositol kinases) [39–53]. These interactions rely on the specific recognition and binding via the SH2 and SH3 domains [33, 54]. Many oncogenes, receptors, receptor ligands and other stimuli are proposed to induce Crk/CRKL complex formation, which links Crk/CRKL with a number of development and tumorigenesis-related signaling pathways [33]. For example, the FGF and VEGF signaling pathways regulated by Crk function in cell proliferation, migration, and survival [55–57]. CRKL modulates EGFR inhibitor resistance in small cell lung cancers and is essential in FGF8 signaling in a mouse disease model [58, 59].

CRKL is tightly linked to leukemia via its binding partners BCR-ABL and TEL-ABL [33, 60]. BCR-ABL is well known to phosphorylate CRKL which plays a role in fibroblast transformation by binding to other adaptor proteins [61–63]. The first SH3 domain of CRKL and a proline-rich region in the C-terminal tail of the ABL kinase mediated the direct interaction of CRKL and BCR-ABL. *CRKL* is overexpressed in various types of human cancer and can induce cancer cell proliferation and invasion [64–67]. In addition, CRKL was demonstrated to be an oncoprotein contributing to malignant cell growth and chemoresistance and promoting cancer cell invasion through a Src-dependent pathway [68].

Key kinases and adaptors in signaling transduction pathways are known to regulate gene transcription [69]. Interestingly, it is emerging that kinases in signal transduction pathways can also modulate the phosphorylation state of SR proteins which are key regulators of alternative splicing [70–74]. Regulation of alternative splicing by key kinases and adaptor proteins might represent a general role in coordinating the cell responses to external and internal signals. Recently, a number of such proteins were reported to be associated with mRNAs in living cells, including CRKL, indicating a previously unknown regulatory mechanisms of these signaling proteins [75]. Nevertheless, it remains unclear whether signaling adaptors such as CRKL could regulate alternative splicing.

In this study, we analyzed the expression level of *CRKL* in 305 cervical cancer tissue samples available in TCGA database, showing a significant increased expression in Stage I cancer samples (Stage I, the carcinoma is strictly confined to the cervix without invasion). We then selected 40 cancer samples with 20 showing high *CRKL* expression and 20 showing low, which were analyzed for the potential impact of CRKL on alternative splicing regulation of cancer transcriptome. We further explored the potential function of CRKL in regulating alternative splicing in HeLa cells using shRNA to knock-down *CRKL* expression. The results confirmed the role of CRKL in promoting cell proliferation in HeLa cell published recently [68], and also showed that CRKL could regulate the alternative splicing of pre-mRNAs from hundreds of genes. We further showed that 94% of CRKL-regulated alternative splicing events detected in HeLa cells could be validated by RT-qPCR approach. Moreover, significantly more CRKL-regulated alternative splicing events detected in HeLa cells were positively than those negatively correlated with the *CRKL* expression level in cervical cancers. These results together support the conclusion that CRKL adaptor protein extensively regulates alternative splicing of many genes which are important in development and tumorigenesis, which expands the functional importance of signaling adaptors in coordinating the dynamic activation of signaling pathways at the alternative splicing level upon cellular responses to various stimuli.

## Methods

### Cell culture and transfections

Human cervical cancer cell lines, HeLa (CCTCC@GDC0009) were obtained from CCTCC (China Center for Type Culture Collection, Wuhan, Hubei, China) in 2017. The HeLa cell line has been authenticated with STR analysis by Cell Bank, Type Culture Collection, Chinese Academy of Sciences (CBTCCCAS), and tested for the free of mycoplasma contamination by the provider. The genomic DNA were purified with Purelink@ Genomic DNA Kits in the Cell Bank. The DNA sample was analyzed in Beijing Microread Genetics Co., Ltd. The sample was amplified with Goldeneye™20A STR Complex Amplification Kit. The profiles STR loci and Amelogenin gene characterized on ABI 3100 Type Genetic Analysis Instrument.

HeLa cells were cultured with 5% CO_2_ at 37 °C in DMEM (Dulbecco’s modified Eagle’s medium), which were with 10% FBS (fetal bovine serum), 100 U/mL penicillin and 100 μg/mL streptomycin. To silencing the expression of CRKL in HeLa cell, we constructed a shRNA-containing plasmid using the vector pGFP-B-RS. The shRNA sense strand against CRKL mRNA sequence was GACCTGTCTTTGCGAAAGCAA. According to the manufacturer’s protocol, shRNA was transfected into HeLa cells using Lipofectamine 2000 (Invitrogen, Carlsbad, CA, USA), which were harvested after 48 h for following RT-qPCR analysis.

### Assessment of the knockdown of *CRKL* by shRNA

We used housekeeping gene *GAPDH* (glyceraldehyde-3-phosphate dehydrogenase) as a control gene for assessing the effects of shRNA targeting CRKL. cDNA synthesis was conducted by standard procedures for following real-time quantification PCR, which was performed on the HieffTM qPCR SYBR® Green Master Mix (Low Rox Plus) (YEASEN, Shanghai, China) to evaluate the knockdown of *CRKL* by shRNA. The information of primers used for RT-qPCR is presented in Additional file 1. The concentration of transcript was then compared with *GAPDH* mRNA level using 2^- ΔΔCT^ method [76] to measure the transcript level of *CRKL*.

### MTT assay

The MTT assay was used to measure cell proliferation. We seeded indicated HeLa cells (1 × 104) in 96-well culture plates with 200 μl of cell growth medium. The vector was transfected into HeLa cells using Lipofectamine 2000 (Invitrogen, Carlsbad, CA, USA) after cells reached at 70% confluence according to the manufacturer’s protocol. Then, the cells were incubated at 37 °C for 48 h. Subsequently, each well of culture plates was added with 25 μl of MTT solution (5 mg/mL), following another 4 h incubation. The supernatant was removed from each well after centrifugation. DMSO was used to dissolved the colored formazan crystals produced from MTT in each well (0.15 mL/well), and the optical density (OD) values were measured at 490 nm.

### RNA extraction and high-throughput sequencing

Total RNA was extracted by the TRIZOL (Ambion) and was further purified with two phenol-chloroform treatments. To remove DNA, the purified RNA was then treated with RQ1 DNase (RNase free) (Promega, Madison, WI, USA) and its quality and quantity were redetermined by measuring the absorbance at 260 nm/280 nm (A260/A280) using Smartspec Plus (BioRad, USA). The integrity of RNA was then verified by 1.5% agarose gel electrophoresis.

We used 10 μg of the total RNA for each sample to preparing directional RNA-seq library. Before that, the polyadenylated mRNAs were concentrated with oligo (dT)-conjugated magnetic beads (Invitrogen, Carlsbad, CA, USA). Then, the concentrated mRNAs were iron fragmented at 95 °C, end repaired and 5′ adaptor ligated with 5′ adaptor. Then, reverse transcription (RT) was performed with RT primer harboring 3′ adaptor sequence and randomized hexamer. The purified cDNAs were amplified and stored at − 80 °C until they were used for sequencing [77]. According to the manufacturer’s instructions, the libraries were prepared for high-throughput sequencing. Illumina HiSeq4000 system was used to collect data from 151-bp pair-end sequencing (ABlife Inc., Wuhan, China).

### RNA-Seq raw data clean and alignment

Raw sequencing reads containing more than 2-N bases were first discarded. Then, the raw reads were trimmed off adaptors and low-quality bases using FASTX-Toolkit (Version 0.0.13). Besides, the short reads less than 16 nt were dropped to gain clean reads, which were subsequently aligned to the GRch38 genome by tophat2 [78] with 4 mismatches. Uniquely mapped reads were ultimately used to calculate reads number and FPKM (paired-end fragments per kilobase of exon per million fragments mapped) value for each gene.

### Differentially expressed genes (DEGs) analysis

The expression level of genes was evaluated using FPKM. We applied the software edgeR [79], which is specifically used to analyze the differential expression of genes, to evaluate the FPKM value and screen out the DEGs (differentially expressed genes) using RNA-Seq data. We analyzed the results based on the fold change (fold change ≥2 or ≤ 0.5) and false discovery rate (FDR < 0.05) to determine whether a gene was differentially expressed.

Using KOBAS 2.0 server [80], Gene Ontology (GO) analyses and enriched KEGG pathway were identified to predict functions of genes and calculate the functional category distribution frequency. The enrichment of each pathway (corrected *p*-value< 0.05) was defined using hypergeometric test and Benjamini-Hochberg FDR controlling procedure.

### Alternative splicing analysis

The ABLas pipeline as described previously [81, 82] was used to define and quantify the ASEs (alternative splicing events) and RASEs (regulated alternative splicing events) between the samples. In brief, detection of seven types of canonical ASEs in each sample was based on the splice junction reads. These ASEs were exon skipping (ES), cassette exon (cassetteExon, CE), alternative 5′splice site (A5SS), alternative 3′splice site (A3SS), mutual exclusive exon skipping (MXE), the MXE combined with alternative polyadenylation site (3pMXE), and with alternative 5′ promoter (5pMXE).

After that, the significant *p*-value was calculated using fisher’s exact test, with the model reads of samples and alternative reads as input data, respectively. We calculated the changed ratio of alternatively spliced reads and constitutively spliced reads between compared samples, which was defined as the RASE ratio. The RASE ratio > 0.2 and p-value < 0.05 were set as the threshold for RASEs detection.

### Reverse transcription qPCR validation of alternative splicing events

To elucidate the validity of ASEs in HeLa cells, quantitative reverse-transcription polymerase chain reaction (RT-qPCR) was performed in this study for some selected RASEs, and normalized with the reference gene GAPDH. The primers for detecting the pre-mRNA splicing are shown in Additional file 1. To quantitatively analyzing the two different splicing isoforms of a specific ASE using a qPCR approach, we designed two pairs of primers to specifically amplify each of these two isoforms after the initial synthesis of the first strand cDNA using random primers. To achieve this specificity, we designed a primer pairing the splice junction of the constitutive exon and alternative exon (Additional file 11). The RNA samples used for RT-qPCR were same to that for RNA-seq. The PCR conditions are consisted of denaturing at 95 °C for 10 min, 40 cycles of denaturing at 95 °C for 15 s, annealing and extension at 60 °C for 1 min. PCR amplifications were respectively performed in triplicate for control and CRKL-KD samples.

### Western blotting analysis

Protein samples were loaded into 10% or 12% SDS-PAGE gels depending on molecular weight and transferred onto 0.45 mm PVDF membranes. The PVDF membranes were then blocked with 5% skim milk (in a buffer containing 10 mM Tris, pH 8.0, 150 mM NaCl, 0.05% Tween 20) for an hour, incubated overnight with primary antibody at 4 °C and then incubated with horseradish peroxidase-conjugated secondary antibody for 1 h at room temperature. Then, membranes were visualized through chemiluminescence. We also have quantitated some of the WB bands by the software Image J. Antibodies: The following antibodies were purchased from commercial sources including anti-AKT2 (Polyclonal Antibody, AB clonal; A0336), anti-phospho-AKT2 (Polyclonal Antibody, Affinity MT; AF3264); anti-CRKL (Polyclonal Antibody, AB clonal; A0511); anti-GAPDH (Polyclonal Antibody, AB clonal; AC001).

### Downloading RNA-seq data of cervical cancer samples

The RNA-seq data of cervical cancer samples were downloaded from TCGA database to analyze the expression of *CRKL* and regulation of alternative splicing in cervical cancer.

## Results

### Expression of *CRKL* is upregulated in cervical cancer, more pronouncedly in early stages, as revealed by TCGA data

**Fig. 1:**
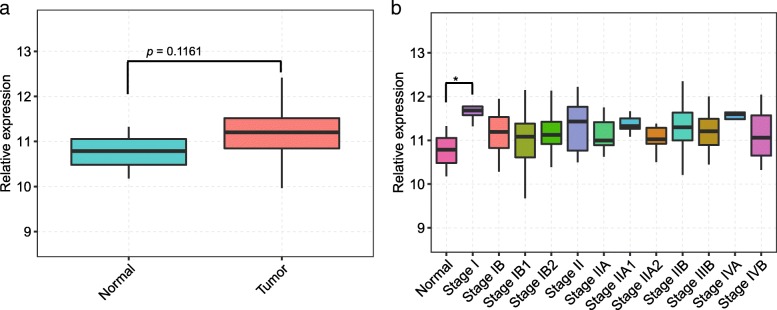
Analysis of *CRKL* expression levels in cervical tumor samples from TCGA database. **a** Plot of the expression of *CRKL* in 3 normal and 305 cervical tumor samples. The data was downloaded from TCGA website. **b** Distribution of expression levels of *CRKL* in normal samples and different stages of cervical tumor samples. Cervical cancer was staged according to standard of international federation of obstetricians and gynecologic oncology (FIGO) stage. Statistical analysis was performed by Student’s t-test: *p < 0.05

Inspired by previous studies on the overexpression of *CRKL* in a small number of cervical cancer samples, we downloaded RNA-seq expression data for all samples available for cervical cancer in TCGA (The Cancer Genome Atlas) database, which included 305 cervical tumor and 3 normal samples. The expression level of *CRKL* was then analyzed. *CRKL* showed higher expression in 305 cervical tumor samples compared with 3 normal tissue samples (Fig. 1a). Among cervical tumor samples, 297 were classified to different stages including I (5), IA (1), IA1 (1), IA2 (1), IB (38), IB1 (77), IB2 (39), II (5), IIA (9), IIA1 (5), IIA2 (7), IIB (42), III (1), IIIA (2), IIIB (43), IVA (9) and IVB (12). The samples in Stage I, II III and IV represented those without distinguishable substages. We further explored the relationship between *CRKL* expression and progression stages of most cervical cancer, with the stages containing at least 5 samples were selected. It was showed that *CRKL* was upregulated in most tumor stages, with a significant upregulation was observed in the stage I samples (Fig. 1b).

### Analysis of potential CRKL-regulated alternative splicing events and genes in cervical cancer clinical samples

**Fig. 2:**
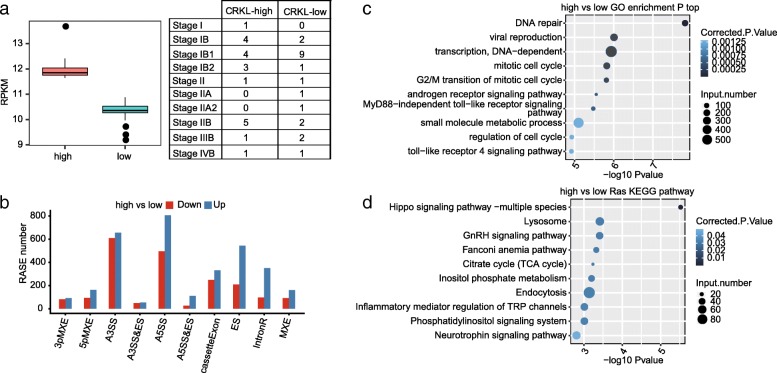
Analysis of potential CRKL-regulated alternative splicing events and genes in cervical cancer clinical samples. **a** Plot of the expression of *CRKL* in two groups of samples with *CRKL* being differently expressed. The samples were grouped based on their CRKL expression level regardless of their pathological stages. **b** Bar plot of the distribution of AS events showing significant difference between the high- and low-CRKL groups of samples. The regulated AS events (RASE) were classified into 10 different types which were detailed in the Methods. The red and blue bars indicate the number of AS events upregulated and downregulated in the high-CRKL groups when compared to the low-CRKL group. **c** The top 10 GO biological process analysis and (**d**) KEGG functional pathway in which the alternative splicing genes were enriched

To uncover the *CRKL*-regulated alternative splicing events (ASEs) in cervical cancer samples, we selected 40 cancer samples with 20 showing high *CRKL* expression and 20 showing low (Fig. 2a). A total of 137 M ± 40 M clean reads per sample were download from TCGA database. Among those, 119 M ± 34 M reads per sample uniquely aligned to the human genome, in which junction reads account for 12.02 to 20.11% (details can be found in Additional file 2). We then used a recently developed ABLas software tool to analyze ASEs from the RNA-seq dataset and detected 33,602 known ASEs and 56,345 novel ASEs, without counting intron retention events. We have validated the efficacy of ABLas software in detecting ASEs from multiple pairs of sample in recently published studies [81, 82].

By applying the stringent cutoff of *p*-value≤0.05, changed AS ratio ≥ 0.2, we identified 5265 high-confidence regulated ASEs (RASEs) that were associated with the *CRKL* expression level in these 40 clinical samples (Fig. 2b). These data suggested that *CRKL* extensively regulated ASEs in cervical carcinoma. Genes harboring *CRKL*-regulated ASEs were highly enriched for DNA repair, viral reproduction, DNA transcription, mitotic cell cycle (G2/M), androgen receptor signaling pathway and toll-like receptor signaling pathway (GO biological process terms, Fig. 2c). Enriched KEGG pathways (*p*-value greater than 0.05) included those involved in Hippo signaling pathway, GnRH signaling pathway, Fanconi anemia pathway, phosphatidylinositol signaling system, neurotrophin signaling pathway, metabolism and inflammatory related function (Fig. 2d). These results together indicate that the potential *CRKL*-regulated ASEs could play a large role in cervical tumorigenesis. Because cancer tissues are complicated in cell types and deregulated genes, these potential *CRKL*-regulated ASEs could be complicated by the contribution of other factors.

### Knock-down of *CRKL* expression in HeLa cell reduce cell proliferation

**Fig. 3:**
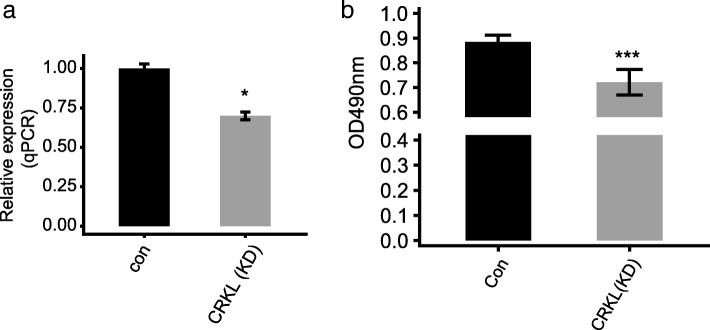
Knockdown of *CRKL* in HeLa cells and its effect on cell proliferation. **a** The expression level of *CRKL* in HeLa cells after transiently transfected with *CRKL* specific shRNA or control vector, detected through RT-qPCR. **b** Cell proliferation analysis. Proliferation of control cells and treated HeLa cells were determined by luminescence read on a Luminometer after adding Cell Titer Glo reagent

To explore whether CRKL regulates alternative splicing at the cell level, we decided to construct a functional cell model. In light of previous study and the CRKL-regulated functional pathway in mitotic cell cycle, we predicted that *CRKL* might regulate the proliferation of the major cell types in cervical cancer. To this end, we knocked down *CRKL* by shRNA in HeLa cells (Fig. 3a and Additional file 3, sequencing information of shRNA was shown in Methods), derived from a cervical cancer patient, and analyzed the cell proliferation rate. Cell proliferation in shRNA treatment group was declined when compared with the control (Fig. 3b), consistent with a possible role of *CRKL* gene in facilitating the proliferation of tumor cells. Our hypothesis and results were consistent with previous report as well [68].

### shCRKL resulted in some transcriptional difference

**Fig. 4:**
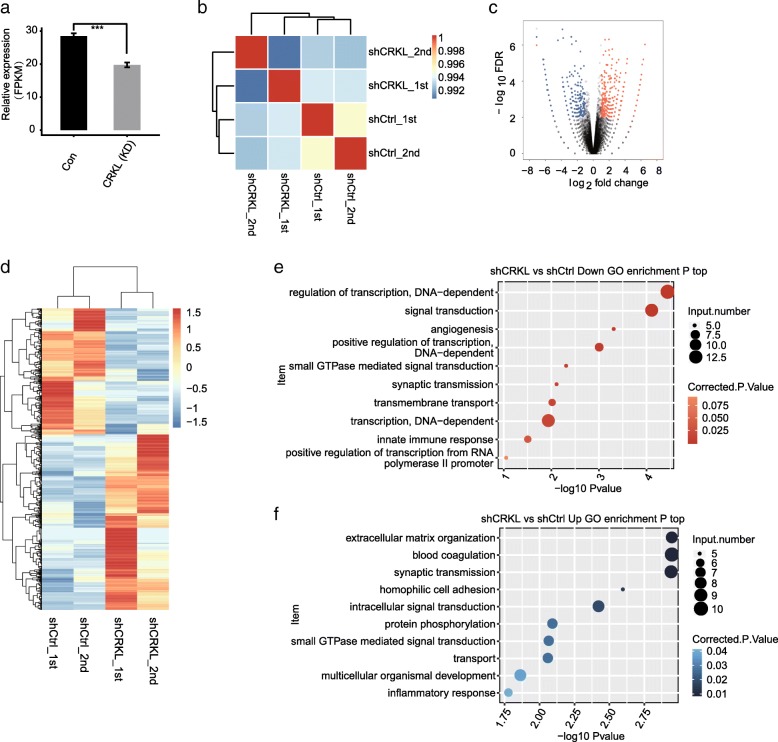
RNA-seq analysis of the change of gene expression in response to the *CRKL* knock-down. **a** *CRKL* expression values quantified by RNA sequencing data. FPKM values were calculated as that has been explained in Methods. **b** Heat map shows the hierarchically clustered Pearson correlation matrix resulting from comparing the transcript expression values for control and *CRKL* shRNA-treated samples. **c** Detection of the *CRKL* regulated genes on the volcano plots, differentially up-regulated genes (FC ≥ 1.5, *P*-value< 0.01) are labeled red, whereas differentially down-regulated (FC ≤ 2/3, P-value< 0.01) are labeled blue. **d** Hierarchical clustering of 837 DEGs in control and shRNA treated samples. Expression values (FPKM) are log2-transformed and then median-centered by each gene. (E-F) The top 10 representative GO Biological Process terms of CRKL-upregulated and downregulated genes

With the purpose of investigating the CRKL-mediated transcriptional regulation, RNA-seq experiments was carried out. We constructed four cDNA libraries prepared from above control and *CRKL* knockdown cells (two biological replicates), which were sequenced on the Illumina HiSeq4000 platform to produce 150 nucleotide paired-end reads per sample. After removing adaptors and contaminating sequences, we obtained a total of 78.3 M ± 4.6 M high-quality reads per sample (details can be found in Additional file 4). An average of 65.3 M ± 4.9 M paired-end reads per sample were then aligned to the human GRCH38 genome and about 94.67–95.34% were uniquely aligned. To compare the gene expression patterns across individuals, we reassessed gene and transcript quantification with Cufflinks [83]. We calculated expression values in units of fragments per kilo base of exon model per million fragments mapped (FPKM) and the expression results for 28,944 genes were yielded from RNA-seq (details can be found in Additional file 5). In addition to RT-qPCR assessment (Fig. 3a), effective knockdown of *CRKL* was further confirmed in parallel RNA-seq analysis (Fig. 4a). FPKM values for all 28,944 genes were used to calculate a correlation matrix based on Pearson’s correlation coefficient. The diagonal of the heat map showed the Pearson correlation between *CRKL*-KD and control cells, where the correlation matrix was symmetric and two biological replicates were highly correlated (Fig. 4b).

Based on above RNA-seq data obtained from *CRKL*-KD cells and control, we then explored genes potentially regulated by *CRKL* at the transcriptional level. Differentially expressed genes (DEGs) between the *CRKL*-KD and control cells were identified using edgeR [79]. Only 130 DEGs were identified when the cutoff was set as fold change (FC) ≥2 or ≤ 0.5 and a 5% false discovery rate (FDR), indicating that shCRKL resulted in a small transcriptional difference. We then adjusted the criteria to FC ≥ 1.5 or ≤ 2/3, *p*-value < 0.01 and identified 837 DEGs, with 487 up-regulated and 350 down-regulated genes respectively (details can be found in Additional file 6). The DEGs related to *CRKL* KD were displayed in a volcano plot (Fig. 4c). Heatmap analysis of the expression patterns of the DEGs in RNA-seq samples showed a high consistency of the CRKL-mediated transcription in both data sets (Fig. 4d).

To reveal the potential biological roles of these DEGs, we subjected all 837 DEGs to GO and KEGG annotation. On the base of the cutoff criterion, the upregulated and downregulated genes were respectively enriched in 60 and 33 GO terms. In the biological process terms of analysis, the upregulated genes in the *CRKL*-KD cells mainly enriched in extracellular matrix organization, blood coagulation, synaptic transmission, signal transduction, and protein phosphorylation (Fig. 4e). The downregulated genes mostly related to regulation of transcription, signal transduction, and synaptic transmission (Fig. 4f). The results showed that genes regulated by *CRKL* at the transcriptional level were not enriched in cancer related pathways.

### Transcriptome analysis of CRKL-mediated alternative splicing

To gain an insight of the role of *CRKL* on alternative splicing (AS) regulation, we further used transcriptome sequencing data to explore the *CRKL*-dependent AS events in HeLa cells. A total of 62 M ± 4.6 M uniquely mapped reads were obtained from *CRKL*-KD and control HeLa cells, in which approximately 37.33%~ 40.36% were junction reads (details can be found in Additional file 4). We detected 68.5% of annotated exons (251,598 out of 367,321 annotated exons) when comparing these uniquely mapped reads to the referenced genome annotation and 164,036 annotated and 203,638 novel splice junctions were detected using Tophat2. We then analyzed AS events from the RNA-seq dataset using ABLas software tool (under submission) to investigate the changes in AS occurrence. We detected 20,618 known alternative splicing events (ASEs) in the model gene we named in the reference genome, and 63,479 novel ASEs, excluding intron retention (IR) (details can be found in Additional file 7).

**Fig. 5:**
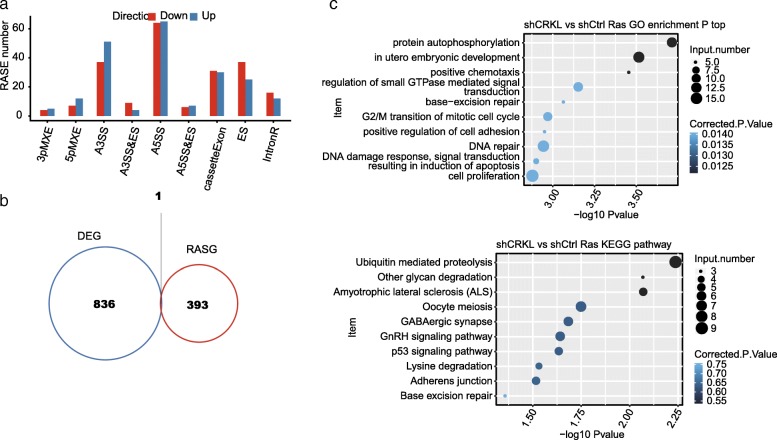
Identification and functional analysis of CRKL-regulated alternative splicing events. **a** Classification of different AS types regulated by CRKL protein. **b** Venn diagram shows the result of overlap analysis between CRKL-regulated differentially expressed genes (DEG) and alternative splicing gene (RASG). **c** The top 10 GO biological process analysis and KEGG functional pathway of the alternative splicing genes

By applying a stringent cutoff of *p*-value ≤0.05, changed AS ratio ≥ 0.2 (See Methods), we identified 417 high-confidence regulated alternative splicing events (RASEs) (details for RASEs can be found in Additional file 8). A majority of RASEs included alternative 5′splice site (A5SS, 129 events), alternative 3′splice site (A3SS, 88 events), exon skipping (ES, 62 events) and cassette exon (CE, 61 events) (Fig. 5a). The data suggested that *CRKL* globally regulates ASEs in HeLa cells. Except that the changes in AS events could be simply attributed to transcription regulation, we also analyzed the expression in transcriptional level of RASGs, genes found to be alternatively spliced differently between CRKL-KD and normal samples. The results showed that there were hardly any significant regulated transcript levels in RASGs (Fig. 5b).

It further revealed that these genes regulated by *CRKL* were highly enriched for the protein autophosphorylation, embryonic development, mitotic cell cycle, DNA repair and cell proliferation (GO biological process terms, Fig. 5c, top panel). Enriched KEGG pathways (p-value greater than 0.05) included those involved in ubiquitin mediated proteolysis, glycan degradation, p53 signaling pathway and Base excision repair (Fig. 5c, bottom panel) (details can be found in Additional file 9). It is interesting to find that a number of GO biological pathways enriched by *CRKL*-regulated alternative splicing in HeLa cell were similar to those in cervical cancer samples (Fig. 2).

It has been reported recently that overexpressed CRKL promotes the phosphorylation of AKT [68, 84]. AKT is a serine-threonine (SR) protein kinase and regulates SR protein kinase activity [74, 85, 86]. We then performed western blot analysis, which showed that the expression level of CRKL was positively correlated with the phosphorylation level of AKT2 (Additional file 10).

### Validation of CRKL-regulated alternative splicing of cancer-related genes in HeLa cells

**Fig. 6:**
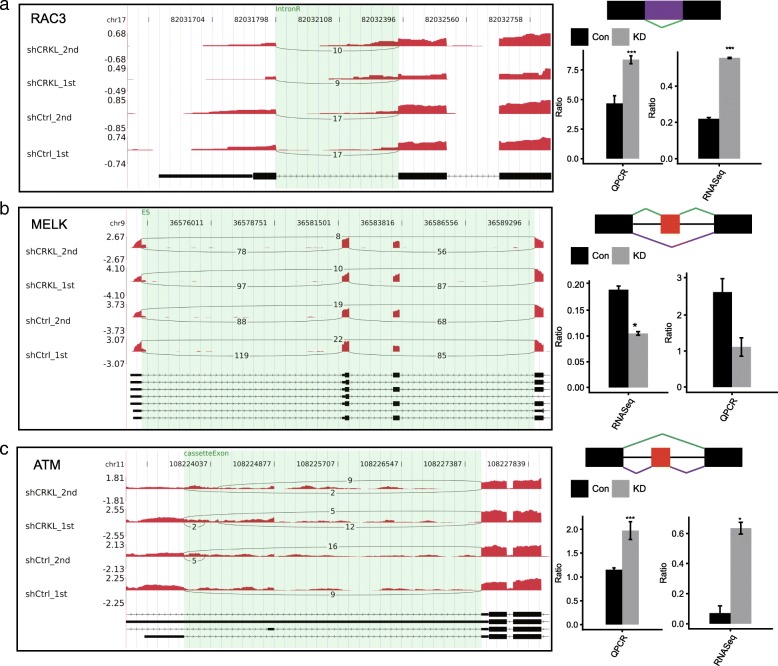
Validation of CRKL-regulated alternative splicing events in cancer related genes. (**a**) ASEs in *RAC3*. (**b**) ASEs in *MELK*. (**c**) ASEs in *ATM*. IGV-sashimi plots show AS changes occurred in *CRKL*-KD cells and control (Left panel) and the transcripts for the gene are shown below. The schematic diagrams depict the structures of ASEs, AS (purple line) and Model (green line). The exon sequences are denoted by boxes and intron sequences by the horizontal line (Right panel, top). RNA-seq quantification and RT-qPCR validation of ASEs are shown in the bottom of right panel. The altered ratio of AS events in RNA-seq were calculated using the formula: AS junction reads / (AS junction reads + Model junction reads); while the altered ratio of AS events in RT-qPCR were calculated using the formula: AS transcripts level /Model transcripts level

To validate the ASEs of cancer related genes regulated by CRKL by a different method, we selected 43 ASEs to perform RT-qPCR experiment. PCR primer pairs (details can be found in Additional file 1) could be designed to amplify the two different splicing isoforms in the same ASE event for 39 ASEs (the design of primers of exon skipping was shown in Additional file 11). Out of these 39 tested events, 34 ASEs validated by RT-qPCR were consistent with the RNA-seq results, including 14 IR, 6 A5SS, 5 A3SS, 4 ES, 3 CE, 1 MXE (mutually exclusive exons) and 1 A5SS&ES (A5SS and ES occur simultaneously). Figure 6 and Additional file 11 show 26 alternative splicing events of them, which locate in 25 relevant genes (*MELK*, *RAC3*, *ATM*, *NTHL1*, *ADAM8*, *SIN3A*, *PTK2B*, *TRAIP*, *CRY1*, *VEGFA*, *TSC2*, *CLK1*, *ERCC6*, *UHRF1*, *UBE2A*, *EPS15*, *RACGAP1*, *CDC16*, *ERCC1*, *ITSN1*, *APC*, *SCRIB*). Most of these genes are kinases, adaptor proteins or transcriptional regulators directly or indirectly involved in tumorigenesis and tumor progression. The results confirmed that CRKL-regulated ASEs identified by ABLas analysis of RNA-seq results were confident.

### CRKL-regulated alternative splicing events in HeLa cells were similarly regulated in a CRKL-dependent manner in cervical cancers samples

**Fig. 7:**
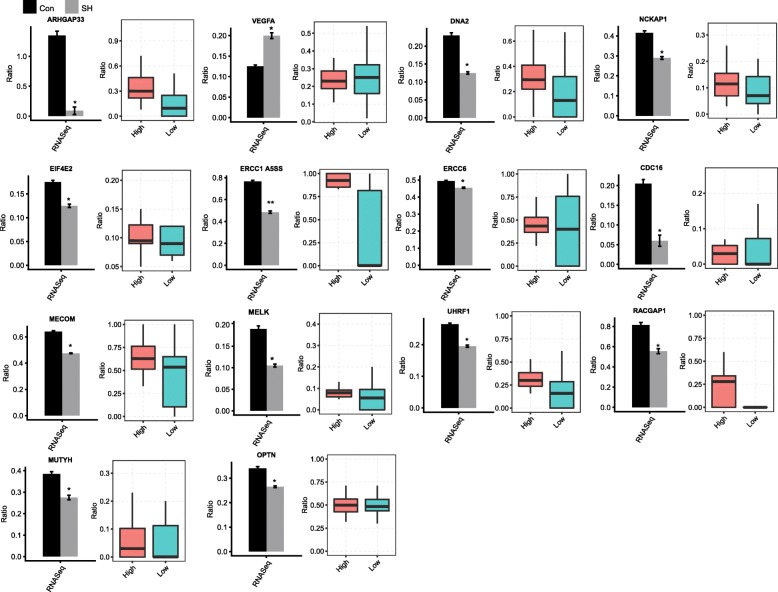
Analysis of CRKL-regulated alternative splicing events in HeLa cells in cervical cancers samples. The ASEs change in same direction responded to *CRKL* expression levels in 40 cervical tumor samples and HeLa cells. RNA-seq quantification of ASEs detected in 40 cervical tumor samples and HeLa cells were respectively shown in box plots (Right panel) and bar plots (Left panel)

We then sought to study how the CRKL-regulated ASEs revealed by RNA-seq in HeLa cells were also regulated by *CRKL* expression in cervical cancer samples. We analyzed the alternative splicing patterns of the 43 CRKL-regulated ASEs which were used for RT-qPCR validation in the RNA-seq data of 40 cervical tumor samples with 20 showing high *CRKL* expression and 20 showing low (Fig. 2). Out of these 43 ASEs, 27 of them were detected in the both *CRKL*-high and *CRKL*-low groups. We compared the CRKL-dependence of these 27 ASE detected both in clinical samples and HeLa cells, showing that 14 of them responded to *CRKL* expression levels in cervical cancer in the same direction as in HeLa cells and only 5 of them showing an apparently opposite response (Fig. 7 and Additional file 12). The alternative splicing event of ATM specifically regulated in HeLa cells was not significant enough to be identified in the clinical samples. However, some other ASEs in ATM were identified, and one of them was differentially spliced between the high and low-CRKL group (Additional file 12). These data indicated that *CRKL* gene might play important roles in cervical tumorigenesis by regulating alternative splicing of important cancer-related genes.

## Discussion

CRKL is a signaling adaptor protein containing SH2 and SH3 domains, which can connect the activated cell-surface receptors with down-stream effectors (kinases) in signaling pathways via mediating molecular interactions [33, 54]. Many oncogenes, receptors, receptor ligands and other stimuli are proposed to link Crk/CRKL with a number of development and tumorigenesis-related signaling pathways, such as FGF, VEGF and EGFR signaling pathways [55–59]. CRKL is overexpressed in a number of types of human malignant tumors, including cervical cancer, lung cancer, breast cancer, gastric cancer, and pancreatic carcinoma. It plays crucial roles in tumorigenesis and cancer progression [65–68, 87]. Regulation of gene transcription and alternative splicing by key kinases and adaptors protein in signaling transduction pathways has been extensively studied [69–74]. A number of such proteins were reported recently to be associated with mRNAs in living cell, including CRKL, indicating a previously unknown regulatory mechanism of these signaling proteins [75]. Nevertheless, it remains unclear whether signaling adaptors such as CRKL could regulate alternative splicing. In the present study, we performed experiments to identify what role CRKL plays in cervical carcinoma and explore whether CRKL could regulate alternative splicing.

We analyzed the expression level of *CRKL* in 305 cervical cancer tissue samples and 3 normal samples by referring to the RNA-seq data available from TCGA database and found a significant increased expression in cervical tumor, especially in Stage I cancer samples (Fig. 1). What cause that *CRKL* has highest expression in Stage I tumor need to be further explored. We then selected 40 cancer samples with 20 showing high *CRKL* expression and 20 showing low, which were analyzed for the potential impact of CRKL on alternative splicing regulation of cancer transcriptome. Alternative splicing of pre-mRNAs from 461 genes, which were enriched in DNA repair, mitotic cell cycle and a number of signaling pathways, were shown to be correlated with the CRKL expression level.

In order to explore whether CRKL is directly involved in regulating alternative splicing in HeLa cells, we established *CRKL*-knockdown (KD) cells by transient transfection of *CRKL*-shRNA and performed cell proliferation experiment. A significant decrease in cell proliferation level in *CRKL*-KD HeLa cells confirmed the role of CRKL in promoting cell proliferation in HeLa cell published recently [68] (Fig. 3). In addition, RNA-seq analysis on *CRKL*-KD and control HeLa cells showed that CRKL could extensively regulate alternative splicing of pre-mRNA from hundreds of genes, which enriched in protein autophosphorylation, embryonic development, DNA repair, mitotic cell cycle, and cell proliferation (Fig. 4). These functional pathways that CRKL-regulated alternative splicing events enriched in are similar as those in cervical cancer samples (Fig. 2). This indicated that the effect of CRKL on alternative splicing might be significantly related to tumorigenesis in cervical cancer. More importantly, we showed that 34 (87%) of CRKL-regulated alternative splicing events detected in HeLa cells could be validated by RT-qPCR approach. SR proteins are well known splicing factors extensively regulate alternative splicing [74, 84]. We and another group have demonstrated that CRKL expression level regulates the phosphorylation of an SR protein AKT (Additional file 10) [68]. These results together suggested that CRKL is directly involved in alternative splicing regulation, and CRKL might achieve this regulation via its positive regulation of AKT2 activity.

Furthermore, we reported that more than a half of the qPCR-validated CRKL-regulated ASEs detected in HeLa cells were also correlated with the *CRKL* expression level in cervical cancers (Fig. 7 and Additional file 12). We noticed that the expression difference between the CRKL-high and CRKL-low samples was relatively small, this small difference could at least partially explain the relative low correlation of the RASEs between HeLa cells and cervical tumor samples.

Here we noted that validated alternative splicing events regulated by CRKL mostly located in genes encoding kinases or adaptor proteins in various signaling pathways or transcription regulation factor, including *RAC3* (Rac Family Small GTPase 3), *PTK2B* (Protein Tyrosine Kinase 2 Beta), *MELK* (Maternal Embryonic Leucine Zipper Kinase), *ATM* (ATM Serine/Threonine Kinase), *TSC2* (Tuberous Sclerosis 2, a tumor suppressor), *EPS15* (Epidermal Growth Factor Receptor Pathway Substrate 15), *RACGAP1* (Rac GTPase Activating Protein 1), *APC* (WNT Signaling Pathway Regulator), *TRAIP* (TRAF Interacting Protein), *SIN3A* (SIN3 Transcription Regulator Family Member A), *CHEK2* (checkpoint kinase), *EIF4E2* (Eukaryotic Translation Initiation Factor 4E Family Member 2) and *UBE2A* (Ubiquitin Conjugating Enzyme E2 A) (Fig. 6 and Additional file 11). Meanwhile, these genes generally involved in tumorigenesis functions including cell proliferation, migration and apoptosis.

*RAC3* encodes a GTPase which is a member of the RAS proto-oncogene superfamily of small GTP-binding proteins. Studies have reported that its related pathways, ERK and RAC signaling, are key regulators in leukocyte and cancer cell migration [88] and *RAC3* was further proved to regulate cell proliferation, differentiation and migration in several cancers [89–91]. More interestingly, *RAC1* as the paralog of *RAC3* was reported to play an important role in cervical cancer progression [92]. *CRKL* depletion significantly alters the retention of variable introns of *RAC3* (Fig. 6), which was changed in the opposite in clinical samples (Additional file 12). This underlines that the functional mechanism of RAC3 in cervical cancer sample maybe need to be further investigated. The *CRKL*-dependent alternative splicing of *APC* and *SCRIB* resulted from the use of a cryptic donor site in respective intron and generate a changed isoform in *CRKL*-knockdown cells (Additional file 11). They both function in tumor suppression pathways involved in cell proliferation, migration and apoptosis [93, 94], which could be affected by their altered isoforms.

Several CRKL-regulated alternative splicing events involved in genes encoding protein kinase, such as *PTK2B*, *MELK*, *TSC2* and *ATM*, which play roles in different signaling pathways or cellular processes. The protein tyrosine kinase PTK2B involved in Ca^2+^-induced regulation of ion channel and MAP kinase activation [61, 95], which has underlying relationship with cervical cancer [96]. *MELK* encodes a protein Serine/Threonine kinase which plays a role in cell proliferation and carcinogenesis [97, 98] and TSC2 as a protein phosphatase regulating mTOR and downstream signaling [99]. *CRKL* depletion significantly alters the retention of variable introns of *PTK2B* and *TSC2* (Additional file 11), and the inclusion of variable exons of *MELK* (Fig. 6), and these tumorigenesis involving genes might then affect development or progression of cervical carcinoma. ATM is emerging as a serine/threonine protein kinase, which belongs to the PI3K/PI4K family and acts as a DNA damage sensor activating checkpoint signaling upon double strand breaks (DBSs) [100]. This is an important cell cycle checkpoint kinase regulating a wide variety of downstream proteins [101, 102], including tumor suppressor proteins p53 and BRCA1, checkpoint kinase CHK2, checkpoint proteins RAD17 and RAD9, oncogenic protein MDM2, and DNA repair protein NBS1. By phosphorylating these substrates, ATM responds swiftly and vigorously to DBSs and affects specific processes in which these proteins are involved. The AS regulation of *ATM* might affect its response functions to DBSs in the process of carcinogenesis (Fig. 6, and Additional file 12).

The alternative spliced *BCL2L1* can modulate cell apoptosis to escape from cell death in cancer, which is critical for tumorigenesis [103]. *CRKL*-depletion regulates alternative splicing to produce shorter isoforms of BCL2L1 which was reported to function as apoptosis activator (Additional file 11). This result indicates that CRKL could contribute to tumorigenesis via regulating the alternative splicing of *BCL2L1*, an inhibitor of cell death. In addition, CRKL regulated alternative splicing of genes encoding proteins function in various cellular process. For example, *SIN3A* as a transcriptional regulator, *UHRF1* as epigenetic regulatory factors, *EPS15* as epidermal Growth Factor Receptor Pathway Substrate, *RACGAP1* as a GTPase-activating protein (GAP), *CDC16* as a component of the anaphase promoting complex/cyclosome (APC/C) and *TUBG2* as a tubulin were all proved to be targeted (Additional file 11), which altogether influence the way CRKL regulates cervical cancer.

## Conclusion

In this study, we have successfully applied RNA-seq technology to demonstrate CRKL regulation of alternative splicing, which is consistent with its reported role as a signaling adaptor, a kinase and a mRNA-associated protein. We showed that, both in HeLa cells and cervical tumor clinical samples, CRKL regulates the alternative splicing of genes which are critical in tumorigenesis and cancer progression. Our results underline that the well-known signaling adaptor protein CRKL might integrate the external and internal cellular signals and coordinate the dynamic activation of cellular signaling pathways including alternative splicing regulation. Further study of CRKL-regulated alternative splicing should contribute to a precise understanding of signaling networks directing tumorigenesis, and potentially CRKL-targeted therapies.

## Additional files

Additional file 1: Primers sequence (xlsx 13 kb). Information of primers used in RT-qPCR experiments. (XLSX 12 kb)

Additional file 2: RNA-seq reads of cervical cancer samples (xlsx 13 kb). Summary of RNA-seq reads of cervical cancer samples obtained from TCGA database used in alternative splicing analysis. (XLSX 12 kb)

Additional file 3: Detection of the transfection efficiency (PDF 2423 kb). Green fluorescence detection of the transfection efficiency. GFP expression plasmid was co-transfected with the scramble control shRNA (left) and CRKL-targeted shRNAs (right). (PDF 2422 kb)

Additional file 4: RNA-seq reads of HeLa cells samples (xlsx 12 kb). Summary of RNA-seq reads of HeLa cells samples used in alternative splicing analysis. (XLSX 11 kb)

Additional file 5: Expressed gene number in HeLa cells (xlsx 10 kb). Statistics of expressed gene number from RNA-seq data of HeLa cells samples. (XLSX 9 kb)

Additional file 6: CRKL-KD vs Ctrl DEGs (xlsx 114 kb). Information of DEGs (differently expressed genes) between CRKL-knockdown and control samples of HeLa cells. (XLSX 113 kb)

Additional file 7: Alternative splicing events (xlsx 11 kb). Statistics of various types of alternative splicing events detected in CRKL-knockdown and control samples of HeLa cells. (XLSX 10 kb)

Additional file 8: shCRKL_vs_Ctrl_RAS_p0.05. Information of RASEs (regulated alternative splicing events) between CRKL-knockdown and control samples of HeLa cells. (XLSX 136 kb)

Additional file 9: RAS GO enrichment and KEGG pathway (xlsx 45 kb). GO and KEGG pathway enrichment of RASEs (regulated alternative splicing events) between CRKL-knockdown and control samples of HeLa cells. (XLSX 44 kb)

Additional file 10: Analysis of kinase activity of AKT2 in HeLa cells with different expression of CRKL (PDF 909 kb). The expression level of AKT2 and P-AKT2 in HeLa cells with high-expression of CRKL (CRKL-high) and low-expression (CRKL-low) groups were investigated by western blotting analysis. Each group has two biological replicates. (PDF 908 kb)

Additional file 11: Validation of ASEs in cancer related genes regulated by CRKL (PDF 1106 kb). The schematic diagrams depict the structures of ASEs, AS (purple line) and Model (green line). The exon sequences are denoted by boxes and intron sequences by the horizontal line (Top panel). RNA-seq quantification and RT-qPCR validation of ASEs are respectively shown in the left and right of the bottom panel. The altered ratio of AS events in RNA-seq were calculated using formula in Fig. 6. The primer pairing the splicing junction of the constitutive exon and alternative exon for RT-qPCR validation was shown as the arrows above the boxes or below on the bottom of the figure. Green arrow represents the right primer pairing the splice junction of constitutive exon and purple arrow represents the alternative, and black is the sharing former primer. (PDF 1105 kb)

Additional file 12: Analysis of CRKL-regulated alternative splicing events in HeLa cells in cervical cancers samples (PDF 6517 kb). RNA-seq quantification of ASEs detected in 40 cervical tumor samples and HeLa cells were respectively shown in box plots (Right panel) and bar plots (Left panel). (A) The ASEs change in opposite direction responded to *CRKL* expression levels in 40 cervical tumor samples and HeLa cells. (B) The ASEs without change in clinical samples with different *CRKL* expression levels. (C) ASEs in ATM were identified to be differentially spliced between the high and low-CRKL group. This ASE are different from the one detected in HeLa cells. IGV-sashimi plots show AS changes occurred in *CRKL*-KD cells and control (Left panel) and the transcripts for the gene are shown below. The schematic diagrams depict the structures of ASEs, AS (purple line) and Model (green line). The exon sequences are denoted by boxes and intron sequences by the horizontal line (Top panel). RNA-seq quantification of ASEs are respectively shown in the bottom panel. The altered ratio of AS events in RNA-seq were calculated using formula in Fig. 6. (PDF 6516 kb)

## Abbreviations

A3SS: Alternative 3’splice site
A5SS: Alternative 5’splice site
ASEs: Alternative splicing events
CE: Cassette exon
CRKL: CRK like proto-oncogene
ES: Exon skipping
FPKM: Fragments per kilo base of exon model per million fragments mapped
MEX: Mutually exclusive exons
RASEs: Regulated alternative splicing events
RT-qPCR: Quantitative reverse transcription polymerase chain reaction
TCGA: The Cancer Genome Atlas
