# Supplementary figures and images for: CRKL regulates alternative splicing of cancer-related genes in cervical cancer samples and HeLa cell
Source: BMC Cancer. 2019 May 27;19:499. doi: 10.1186/s12885-019-5671-8 (PMC6537309; doi:10.1186/s12885-019-5671-8)

Con

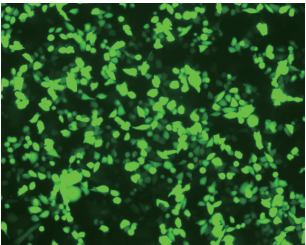

CRKL (KD)

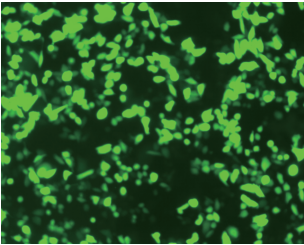

Supplement: Supplementary file 3 — Detection of the transfection efficiency (PDF 2423 kb). Green fluorescence detection of the transfection efficiency. GFP expression plasmid was co-transfected with the scramble control shRNA (left) and CRKL-targeted shRNAs (right). (PDF 2422 kb) [file 12885_2019_5671_MOESM3_ESM.pdf]

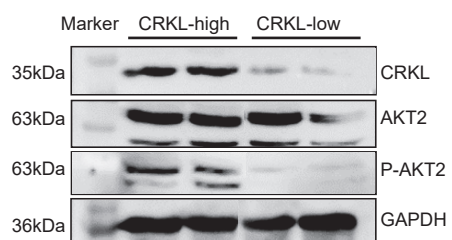

Supplement: Supplementary file 10 — Analysis of kinase activity of AKT2 in HeLa cells with different expression of CRKL (PDF 909 kb). The expression level of AKT2 and P-AKT2 in HeLa cells with high-expression of CRKL (CRKL-high) and low-expression (CRKL-low) groups were investigated by western blotting analysis. Each group has two biological replicates. (PDF 908 kb) [file 12885_2019_5671_MOESM10_ESM.pdf]

Con SH

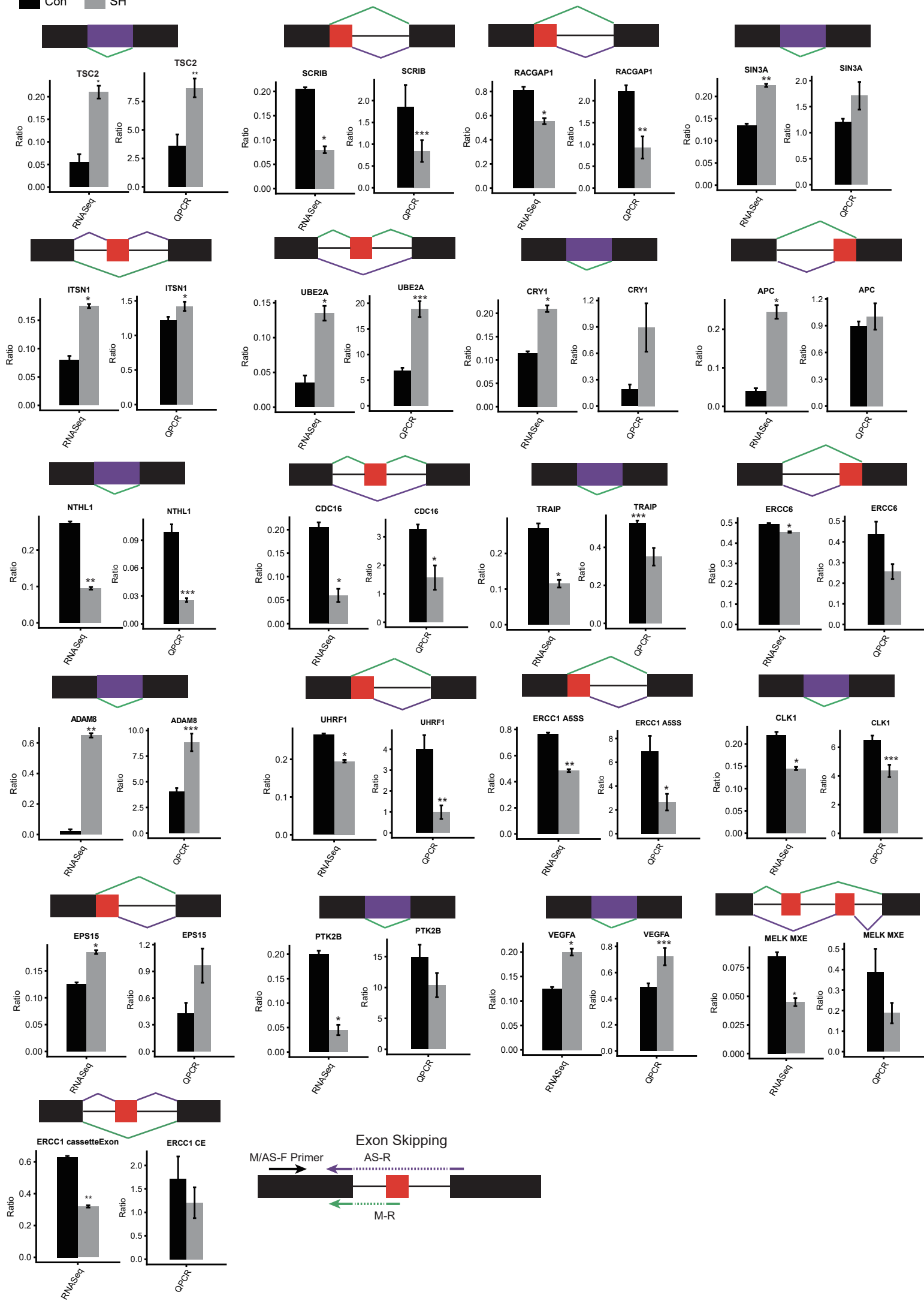

Supplement: Supplementary file 11 — Validation of ASEs in cancer related genes regulated by CRKL (PDF 1106 kb). The schematic diagrams depict the structures of ASEs, AS (purple line) and Model (green line). The exon sequences are denoted by boxes and intron sequences by the horizontal line (Top panel). RNA-seq quantification and RT-qPCR validation of ASEs are respectively shown in the left and right of the bottom panel. The altered ratio of AS events in RNA-seq were calculated using formula in Fig. 6. The primer pairing the splicing junction of the constitutive exon and alternative exon for RT-qPCR validation was shown as the arrows above the boxes or below on the bottom of the figure. Green arrow represents the right primer pairing the splice junction of constitutive exon and purple arrow represents the alternative, and black is the sharing former primer. (PDF 1105 kb) [file 12885_2019_5671_MOESM11_ESM.pdf]

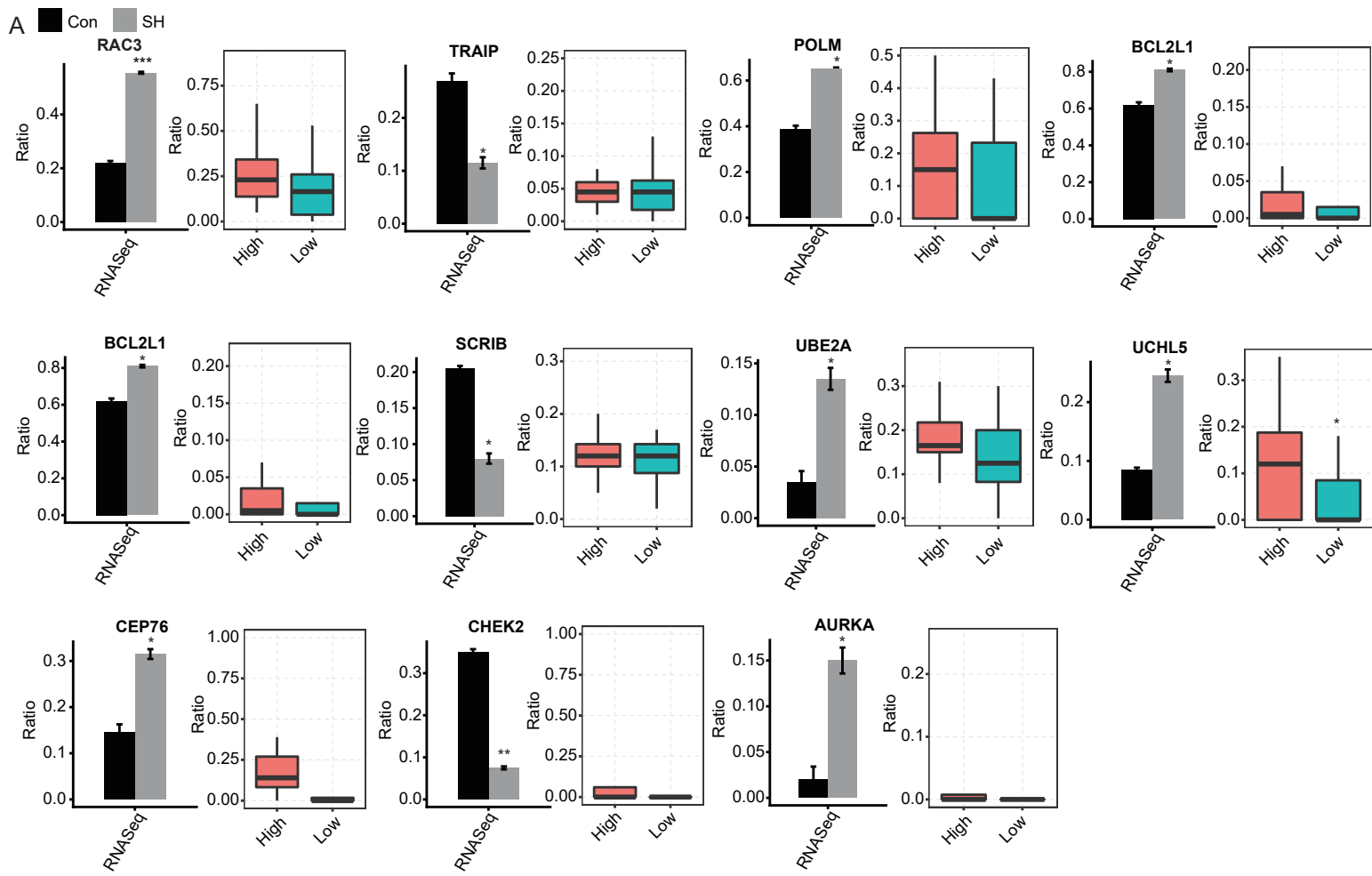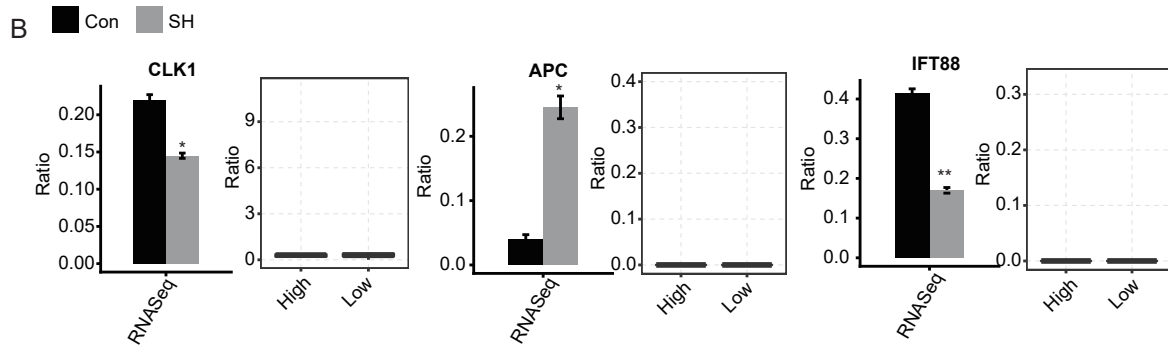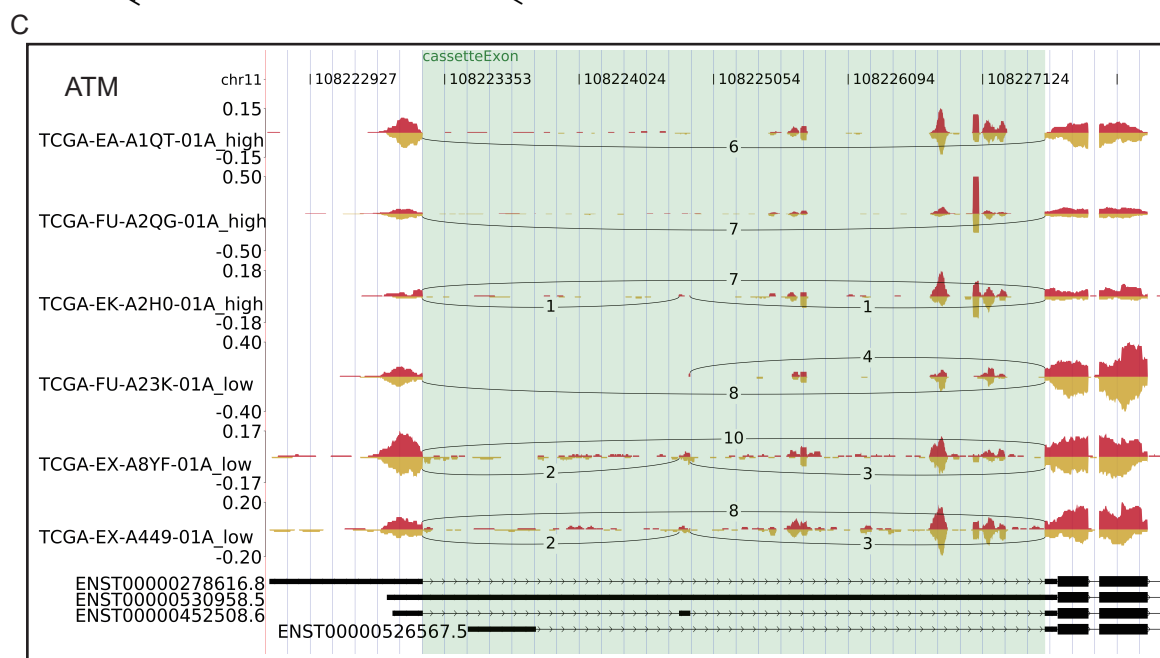

Supplement: Supplementary file 12 — Analysis of CRKL-regulated alternative splicing events in HeLa cells in cervical cancers samples (PDF 6517 kb). RNA-seq quantification of ASEs detected in 40 cervical tumor samples and HeLa cells were respectively shown in box plots (Right panel) and bar plots (Left panel). (A) The ASEs change in opposite direction responded to CRKL expression levels in 40 cervical tumor samples and HeLa cells. (B) The ASEs without change in clinical samples with different CRKL expression levels. (C) ASEs in ATM were identified to be differentially spliced between the high and low-CRKL group. This ASE are different from the one detected in HeLa cells. IGV-sashimi plots show AS changes occurred in CRKL-KD cells and control (Left panel) and the transcripts for the gene are shown below. The schematic diagrams depict the structures of ASEs, AS (purple line) and Model (green line). The exon sequences are denoted by boxes and intron sequences by the horizontal line (Top panel). RNA-seq quantification of ASEs are respectively shown in the bottom panel. The altered ratio of AS events in RNA-seq were calculated using formula in Fig. 6. (PDF 6516 kb) [file 12885_2019_5671_MOESM12_ESM.pdf]
